# Outcomes in Lumbar Fusion Patients Stratified by the Clinical and Radiographic Degenerative Spondylolisthesis (CARDS) Classification System

**DOI:** 10.7759/cureus.54177

**Published:** 2024-02-14

**Authors:** Justin J Turcotte, Jane C Brennan, Parimal Rana, Andrea H Johnson, Chad Patton

**Affiliations:** 1 Orthopedic Research, Anne Arundel Medical Center, Annapolis, USA; 2 Orthopedics, Anne Arundel Medical Center, Annapolis, USA; 3 Orthopedic Surgery, Anne Arundel Medical Center, Annapolis, USA

**Keywords:** patient reported outcomes, transforaminal lumbar interbody fusion (tlif), posterior lumbar fusion, clinical and radiographic degenerative spondylolisthesis (cards, degenerative spondylolisthesis

## Abstract

Background and objective

The Meyerding classification system remains the most common classification system for spondylolisthesis based on the percentages of vertebral translation. However, the majority of patients with degenerative disease fall into Grade 1, limiting its utility in this subset of patients. The Clinical and Radiographic Degenerative Spondylolisthesis (CARDS) classification system provides a simple radiographic framework for classifying degenerative lumbar spondylolisthesis (DLS) patients by incorporating disc height, kyphosis, and anterior translation. The purpose of this study was to evaluate how clinical characteristics, treatments, and outcomes vary across different CARDS groups in patients undergoing one- or two-level lumbar fusion for DLS.

Methods

The patients were classified into one of the following four CARDS groups - Type A: advanced disc space collapse with no evidence of kyphosis; Type B: partially preserved disc space with less than 5.0 mm of translation; Type C: partially preserved disc space with greater than 5.0 mm of translation; and Type D: kyphotic alignment. Univariate analyses were performed to compare demographics, symptoms, clinical outcomes, and Patient-Reported Outcomes Measurement Information System (PROMIS) physical (PH) and mental health (MH) scores across groups.

Results

Ninety-one patients were included in the study. Based on the CARDS classification, there were three (3%) Type A patients, 25 (28%) Type B, 58 (64%) Type C, and five (5%) Type D. No significant differences in baseline demographics, symptom duration, or PROMIS scores were observed across groups. Interbody utilization varied, ranging from 19% in CARDS C (n=11) to 60% in CARDS B (n=15) and D (n=3) patients (p=0.005). Thirty-day clinical outcomes were similar across groups. At an average follow-up of 8.9 months, improvements in PROMIS PH and MH scores and rates of clinically significant improvement were similar across groups.

Conclusions

Based on our findings, patients undergoing lumbar fusion for DLS present with similar demographic and clinical characteristics and experience similar clinical and patient-reported outcomes when stratified using the CARDS classification system. Posterolateral fusion (PLF) can be effective for various radiographic presentations of DLS. Further research is warranted to assess the utility of CARDS in preoperative planning.

## Introduction

Degenerative lumbar spondylolisthesis (DLS) is a relatively common spinal pathology, affecting 6-13% of the adult population [1]. While the vast majority of patients with DLS are asymptomatic, the anterior translation of adjacent vertebral segments can result in symptomatic spinal stenosis causing severe axial and radicular pain [2,3]. Due to the heterogeneous nature of DLS, which may present with a variety of clinical and radiographic characteristics, no universally agreed-upon treatment guidelines exist [4]. Certain large prospective studies have demonstrated that patients failing conservative therapy experience improved outcomes with surgery over continued nonoperative management at two to eight years of follow-up [5-7]; however, there is an ongoing debate regarding the optimal surgical intervention [8-10].

At present, the Meyerding classification system remains the most commonly used method for classifying spondylolisthesis based on the percentage of vertebral translation [11]. The system categorizes slips into five grades: Grade 1 corresponds to 0-25% slippage of the vertebral body, Grade 2 to 25-50%, Grade 3 to 50-75%, Grade 4 to 75%100%, and Grade 5 to greater than 100% (spondyloptosis) [12,13]. However, the majority of patients with degenerative disease fall into the Grade 1 classification, limiting its utility in this subset of spondylolisthesis patients [14]. Furthermore, as the Meyerding system is based on anterior translation, it does not include other clinically relevant radiographic parameters such as disc height and kyphosis [14]. To implement a system addressing the anatomic variations in DLS patients specifically, the Clinical and Radiographic Degenerative Spondylolisthesis (CARDS) classification was developed in 2015 by Kepler et al. [4]. According to this system, DLS is classified into four types: Type A is defined as DLS with “bone on bone” apposition of vertebral endplates; Type B retains partial preservation of the disc space with less than 5 mm anterior translation; Type C retains partial preservation of the disc space with greater than 5 mm translation; and type D DLS demonstrates kyphotic alignment between vertebral levels on ≥1 lateral radiographic view [4,15]. Additionally, a leg pain modifier may be used to subclassify groups based on the absence of leg pain (modifier 0), unilateral leg pain (modifier 1), or bilateral leg pain (modifier 2) [4].

Several studies have demonstrated that the CARDS classification system has high validity, inter- and intra-observer reliability, and is easy to utilize in practice [4,11,16,17]. However, relatively few studies have evaluated differences in clinical characteristics, treatments, and outcomes across CARDS classes so far [11,14,15,18,19]. Hence, this study aimed to evaluate how these factors vary across CARDS groups in patients undergoing posterolateral fusion (PLF) for DLS. Given the heterogeneity of the DLS population, we hypothesized that patient characteristics, surgical treatments, and outcomes would vary across CARDS groups.

## Materials and methods

Patient population

A retrospective review of patients undergoing one- or two-level lumbar fusion, either PLF or PLF and transforaminal lumbar interbody fusion or posterior lumbar interbody fusion (PLF+TLIF/PLIF), by two fellowship-trained orthopedic spine surgeons at a single institution was performed. All patients included in the study were >18 years of age, had a confirmed diagnosis of DLS, and underwent surgery between May 1, 2021 and July 30, 2023. All included patients completed the Patient-Reported Outcomes Measurement Information System (PROMIS) global health instrument, including the physical health (PH) and mental health (MH) components, preoperatively and at a minimum of three months postoperatively.

Spondylolisthesis classification

Preoperative sagittal radiographs were reviewed to classify patients according to the CARDS system. Patients were classified into one of the following four CARDS groups: Type A: advanced disc space collapse with no evidence of kyphosis; Type B: partially preserved disc space with less than 5.0 mm of translation; Type C: partially preserved disc space with greater than 5.0 mm of translation; and Type D: kyphotic alignment pattern [4,14]. Patients with two-level DLS with differing CARDS classifications were classified into the highest group. Patients in the CARDS C group were further subclassified as C(a) (5.0-9.0 mm of translation) and C(b) (greater than 9.0 mm of translation).

Data collection

Patient demographics, comorbidity burden as measured by the American Society of Anesthesiologists (ASA) score, and clinical outcomes were extracted from the electronic medical record by using a structured query language. PROMIS scores and self-reported symptom duration and types were captured during clinic visits as structured data elements and also extracted using structured queries. Minimal clinically important difference (MCID) in PROMIS scores was defined as an improvement of 5 points [1/2 standard deviation (SD)] from baseline to the last follow-up, in alignment with prior studies [20,21]. Surgical details, including the number of operative levels and interbody use, were abstracted manually from the operative notes, and all extracted data points were manually audited to ensure accuracy.

Statistical analysis

Univariate analyses were performed to compare baseline characteristics and outcomes across CARDS groups. One-way analysis of variance (ANOVA) was used to compare continuous and Chi-square test was employed to compare categorical measures across groups. The Fisher’s exact test was performed when the assumptions of Chi-square testing were not met. For the subgroup analyses comparing CARDS B vs. CARDS C patients and CARDS C(a) vs. CARDS C(b) patients, two-sided independent samples t-tests and Chi-square tests were performed. Statistical significance was set at p<0.05. All statistical analysis was performed in RStudio (R Foundation for Statistical Computing, Vienna, Austria).

## Results

In total, 91 patients were included in the study. Based on the CARDS classification, there were three (3%) Type A patients, 25 (28%) Type B, 58 (64%) Type C, and five (5%) Type D. On average, patients were 68.9 years of age and had a BMI of 31.0 kg/m^2^. A total of 67 (74%) patients were female, 22 (24%) were of non-white race, 45 (50%) were current or former smokers, and 51 (56%) had an ASA score of 3 or greater. No significant differences in demographics or comorbidities were observed across CARDS groups (Table 1).

**Table 1:** Patient demographics ASA: American Society of Anesthesiologists; BMI: body mass index; CARDS: Clinical and Radiographic Degenerative Spondylolisthesis; SD: standard deviation

Variables	All patients (n=91)	CARDS A (n=3)	CARDS B (n=25)	CARDS C (n=58)	CARDS D (n=5)	P-value
Age, years, mean ± SD	68.9 ± 10.5	67.3 ± 27.2	67.9 ± 8.5	69.3 ± 10.7	7.4 ± 6.7	0.541
Non-white race, n (%)	22 (24.2)	0 (0)	7 (28.0)	13 (22.4)	2 (40.0)	0.586
Sex, n (%)						0.438
Female	67 (73.6)	2 (66.7)	16 (64.0)	46 (79.3)	3 (60.0)	
Male	24 (26.4)	1 (33.3)	9 (36.0)	12 (20.7)	2 (40.0)	
BMI, kg/m^2^,mean ± SD	31.0 ± 5.8	34.9 ± 3.9	30.9 ± 5.8	30.5 ± 5.8	35.7 ± 6.2	0.214
Smoker (current or former), n (%)	45 (49.5)	3 (100)	11 (44.0)	30 (51.7)	1 (20.0)	0.157
ASA score 3+, n (%)	51 (56.0)	1 (33.3)	16 (64.0)	31 (53.4)	3 (60.0)	0.691

Preoperatively, the average PROMIS PH and MH scores in the whole cohort were 35.4 and 44.0, respectively. A total of 38 (42%) patients presented with symptoms lasting more than two years, 18 (20%) with one to two years, and 35 (39%) with less than one year. The significant majority of patients (n=76, 84%) presented with both axial and radicular symptoms. No significant differences in baseline PROMIS scores or symptom duration were observed across CARDS groups; however, a greater prevalence of axial-only symptoms was observed in the CARDS D group (p=0.016) (Table 2).

**Table 2:** Preoperative PROMIS physical and mental health scores and symptom duration and type P-values <0.05 are shown in bold CARDS: Clinical and Radiographic Degenerative Spondylolisthesis; PROMIS: Patient-Reported Outcomes Measurement Information System; SD: standard deviation

Variables	All Patients (n=91)	CARDS A (n=3)	CARDS B (n=25)	CARDS C (n=58)	CARDS D (n=5)	P-value
Preop PROMIS-physical health, mean ± SD	35.4 ± 6.9	35.7 ± 3.8	36.7 ± 6.8	34.9 ± 7.3	34.9 ± 1.8	0.639
Preop PROMIS-mental health, mean ± SD	44.0 ± 9.6	44.7 ± 12.4	46.7 ± 8.3	42.7 ± 10.3	44.9 ± 3.2	0.460
Symptom duration, years, n (%)						0.442
>2	38 (41.8)	2 (66.7)	11 (44.0)	24 (41.4)	1 (20.0)	
1-2	18 (19.8)	0 (0)	2 (8.0)	14 (24.1)	2 (40.0)	
<1	35 (38.5)	1 (33.3)	12 (48.0)	20 (34.5)	2 (40.0)	
Symptom type, n (%)						
Axial only	6 (6.6)	0 (0)	2 (8.0)	2 (3.4)	2 (40.0)	0.016
Radicular only	7 (7.7)	0 (0)	1 (4.0)	5 (8.6)	1 (20.0)	0.600
Axial and radicular	76 (83.5)	3 (100)	22 (88.0)	49 (84.5)	2 (40.0)	0.048

Assessment of surgical details revealed significant differences across CARDS groups in the proportion of patients undergoing two-level fusion (p=0.036), millimeters of translation (p<0.001), and the proportion of patients undergoing PLF+TLIF/PLIF (p=0.005). Overall, 41% of patients underwent two-level fusion, the average translation was 7.0 mm, and 33% of patients underwent PLF+TLIF/PLIF. Rates of two-level fusion ranged from 20% in CARDS B patients to 80% in CARDS D patients. Average translation ranged from 0 mm in CARDS A patients to 8.8 mm in CARDS C patients. PLF+TLIF/PLIF was most commonly performed in CARDS B (n=15, 60%) and D patients (n=3, 60%), followed by CARDS A (n=1, 33%), and CARDS C (n=11, 19%) (Table 3).

**Table 3:** Surgical details P-values <0.05 are shown in bold CARDS: Clinical and Radiographic Degenerative Spondylolisthesis; SD: standard deviation

Variables	All patients (n=91)	CARDS A (n=3)	CARDS B (n=25)	CARDS C (n=58)	CARDS D (n=5)	P-value
Two-level fusion, n (%)	37 (40.7)	1 (33.3)	5 (20.0)	27 (46.6)	4 (80.0)	0.036
Max translation, mm, mean ± SD	7.0 ± 4.0	0 ± 0	3.6 ± 1.5	8.8 ± 2.9	8.3 ± 8.3	<0.001
Interbody fusion, n (%)	30 (33.0)	1 (33.3)	15 (60.0)	11 (19.0)	3 (60.0)	0.005

During the early postoperative period, no significant differences in clinical outcomes were observed across the CARDS groups. For the entire population, the average hospital length of stay (LOS) was 56.3 hours and 2.0 days. Non-home discharge occurred in four (4%) patients, 14 (15%) returned to the emergency department (ED), seven (8%) were readmitted, and three (3%) underwent reoperation within 30 days of surgery. The average time to patient follow-up was 8.9 months and did not differ across groups. At the last follow-up, the average PROMIS PH score was 42.5 and did differ across groups (p=0.004); the average PROMIS MH score was 48.6 and was similar across groups. On average, PROMIS PH and MH scores improved 7.1 and 4.6 from baseline, with 56 (62%) and 41 (45%) patients experiencing clinically significant improvement on these measures, respectively. No significant differences in PROMIS change scores or MCID achievement rates were observed across CARDS groups (Table 4).

**Table 4:** Postoperative outcomes P-values <0.05 are presented in bold CARDS: Clinical and Radiographic Degenerative Spondylolisthesis; DC: discharge; ED: emergency department; LOS: length of stay; MCID: minimal clinically important difference; PROMIS, Patient-Reported Outcomes Measurement Information System; SD: standard deviation

	All Patients (n=91)	CARDS A (n=3)	CARDS B (n=25)	CARDS C (n=58)	CARDS D (n=5)	P-value
LOS, hours, mean ± SD	56.3 ± 34.2	34.3 ± 21.0	65.3 ± 47.1	53.7 ± 27.3	54.4 ± 34.0	0.576
LOS, days, mean ± SD	2.0 ± 1.4	1.0 ± 1.0	2.4 ± 1.9	1.9 ± 1.2	1.8 ± 1.3	0.404
Non-home DC, n (%)	4 (4.4)	0 (0)	2 (8.0)	1 (1.7)	1 (20.0)	0.188
30-day ED return, n (%)	14 (15.4)	0 (0)	3 (12.0)	11 (19.0)	0 (0)	0.523
30-day readmission, n (%)	7 (7.7)	0 (0)	1 (4.0)	6 (10.3)	0 (0)	0.632
Return to OR, n (%)	3 (3.3)	0 (0)	0 (0)	3 (5.2)	0 (0)	0.623
Postop PROMIS-physical health, mean ± SD	42.5 ± 8.5	41.8 ± 16.1	45.4 ± 6.1	41.7 ± 9.0	37.4 ± 3.9	0.044
Postop PROMIS-mental health, mean ± SD	48.6 ± 9.0	43.4 ± 14.0	52.4± 6.1	47.4± 9.7	46.4 ± 4.4	0.057
Change in pre-postop PROMIS-physical health, mean ± SD	7.1 ± 8.5	6.1± 16.6	8.7 ± 6.3	6.8 ± 9.0	2.5± 5.6	0.403
Change in pre-postop PROMIS-mental health, mean ± SD	4.6 ± 9.0	-1.3 ± 7.6	5.7± 8.2	4.7 ± 9.6	1.4 ± 5.1	0.370
MCID PROMIS-physical health, n (%)	56 (61.5)	2 (66.7)	16 (64.0)	37 (63.8)	1 (20.0)	0.276
MCID PROMIS-mental health, n (%)	41 (45.1)	1 (33.3)	12 (48.0)	27 (46.6)	1 (20.0)	0.665
Follow-up, months, mean ± SD	8.9 ± 6.0	6.5 ± 0.5	8.0± 6.0	9.2± 6.2	11.9 ± 5.5	0.190

In the first subgroup analysis, which compared only CARDS B and CARDS C patients, no significant differences in baseline demographics or comorbidities were observed between groups. CARDS C patients were more likely to undergo two-level fusion (47 vs. 20%, p=0.042) and less likely to undergo PLF+TLIF/PLIF (19 vs. 60%, p<0.001). No significant differences in baseline PROMIS scores, symptom duration, or symptom type were observed between the CARDS B and C patients. CARDS C patients displayed lower levels of postoperative physical health (41.7 vs. 45.4, p=0.028) and mental health (47.4 vs. 52.4, p=0.012) as measured by the PROMIS instruments. However, no significant differences in pre to postoperative change in PROMIS scores or rates of MCID achievement were observed (Table 5).

**Table 5:** Characteristics and outcomes in CARDS B and C patients P-values <0.05 are presented in bold ASA: American Society of Anesthesiologists; BMI: body mass index; CARDS: Clinical and Radiographic Degenerative Spondylolisthesis; DC: discharge; ED: emergency department; LOS: length of stay; MCID: minimal clinically important difference; PROMIS, Patient-Reported Outcomes Measurement Information System; SD: standard deviation

Variables	CARDS B (n=25)	CARDS C (n=58)	P-value
Demographics and comorbidities			
Age, years, mean ± SD	67.9 ± 8.5	69.3 ± 10.7	0.222
Non-white race, n (%)	7 (28.0)	13 (22.4)	0.790
Sex, n (%)			0.231
Female	16 (64.0)	46 (79.3)	
Male	9 (36.0)	12 (20.7)	
BMI, kg/m^2^,mean ± SD	30.9 ± 5.8	30.5 ± 5.8	0.996
Smoker (current or former), n (%)	11 (44.0)	30 (51.7)	0.684
ASA score 3+, n (%)	16 (64.0)	31 (53.4)	0.517
Preop characteristics			
Preop PROMIS-physical health, mean ± SD	36.7 ± 6.8	34.9 ± 7.3	0.228
Preop PROMIS-mental health, mean ± SD	46.7 ± 8.3	42.7 ± 10.3	0.108
Symptom duration, years, n (%)			0.200
>2	11 (44.0)	24 (41.4)	
1-2	2 (8.0)	14 (24.1)	
<1	12 (48.0)	20 (34.5)	
Symptom type, n (%)			
Axial only	2 (8.0)	2 (3.4)	0.742
Radicular only	1 (4.0)	5 (8.6)	0.777
Axial and radicular	22 (88.0)	49 (84.5)	0.938
Surgical details			
Two-level fusion, n (%)	5 (20.0)	27 (46.6)	0.042
Max translation, mm, mean ± SD	3.6 ± 1.5	8.8 ± 2.9	<0.001
Interbody fusion, n (%)	15 (60.0)	11 (19.0)	<0.001
Postop outcomes			
LOS, hours, mean ± SD	65.3 ± 47.1	53.7 ± 27.3	0.612
LOS, days, mean ± SD	2.4 ± 1.9	1.9 ± 1.2	0.367
Non-home DC, n (%)	2 (8.0)	1 (1.7)	0.445
30-day ED return, n (%)	3 (12.0)	11 (19.0)	0.647
30-day readmission, n (%)	1 (4.0)	6 (10.3)	0.600
Return to OR, n (%)	0 (0)	3 (5.2)	0.605
Postop PROMIS-physical health, mean ± SD	45.4 ± 6.1	41.7 ± 9.0	0.028
Postop PROMIS-mental health, mean ± SD	52.4 ± 6.1	47.4 ± 9.7	0.012
Change in pre-postop PROMIS-physical health, mean ± SD	8.7 ± 6.3	6.8 ± 9.0	0.427
Change in pre-postop PROMIS-mental health, mean ± SD	5.7 ± 8.2	4.7 ± 9.6	0.792
MCID PROMIS-physical health, n (%)	16 (64.0)	37 (63.8)	1
MCID PROMIS-mental health, n (%)	12 (48.0)	27 (46.6)	1
Follow-up, months, mean ± SD	8.0 ± 6.0	9.2 ± 6.2	0.153

In the second subgroup analysis, which compared CARDS C(a) (5.0-9.0 mm translation) and CARDS C(b) (greater than 9.0 mm translation) patients, no significant differences in demographics, comorbidities, surgical details, symptom duration, or symptom type were observed between groups. CARDS C(b) patients presented with higher levels of physical health (38.1 vs. 32.5, p=0.002) but similar levels of mental health. No significant differences in clinical or patient-reported outcomes were observed between the C(a) and C(b) groups (Table 6).

**Table 6:** Characteristics and outcomes in CARDS C(a) and C(b) patients P-values <0.05 are presented in bold ASA: American Society of Anesthesiologists; BMI: body mass index; CARDS: Clinical and Radiographic Degenerative Spondylolisthesis; DC: discharge; ED: emergency department; LOS: length of stay; MCID: minimal clinically important difference; PROMIS, Patient-Reported Outcomes Measurement Information System; SD: standard deviation

Variables	CARDS C(a), 5-9 mm translation, (n=33)	CARDS C(b), 9+ mm translation, (n=25)	P-value
Demographics and comorbidities			
Age, years, mean ± SD	70.7 ± 11.2	67.5 ± 9.8	0.099
Non-white race, n (%)	6 (18.2)	7 (28.0)	0.569
Sex, n (%)			0.660
Female	25 (75.8)	21 (84.0)	
Male	8 (24.2)	4 (16.0)	
BMI, kg/m^2^,mean ± SD	31.1 ± 5.7	29.7 ± 5.8	0.456
Smoker (current or former), n (%)	17 (51.5)	13 (52.0)	1
ASA score 3+, n (%)	21 (63.6)	10 (40.0)	0.128
Preop characteristics			
Preop PROMIS-physical health, mean ± SD	32.5 ± 7.6	38.1 ± 5.7	0.002
Preop PROMIS-mental health, mean ± SD	41.1 ± 10.9	44.8 ± 9.4	0.172
Symptom duration, years, n (%)			0.831
>2	14 (42.4)	10 (40.0)	
1-2	7 (21.2)	7 (28.0)	
<1	12 (36.4)	8 (32.0)	
Symptom type, n (%)			
Axial only	2 (6.1)	0 (0)	0.599
Radicular only	1 (3.0)	4 (16.0)	0.204
Axial and radicular	29 (87.9)	20 (80.0)	0.649
Surgical details			
Two-level fusion, n (%)	15 (45.5)	12 (48.0)	1
Max translation, mm, mean ± SD	6.7 ± 1.2	11.5 ± 2.2	<0.001
Interbody fusion, n (%)	5 (15.2)	6 (24.0)	0.608
Postop outcomes			
LOS, hours, mean ± SD	51.6 ± 24.6	56.5 ± 30.9	0.572
LOS, days, mean ± SD	1.8 ± 1.0	2.0 ± 1.3	0.894
Non-home DC, n (%)	1 (3.0)	0 (0)	1
30-day ED return, n (%)	7 (21.2)	4 (16.0)	0.870
30-day readmission, n (%)	3 (9.1)	3 (12.0)	1
Return to OR, n (%)	2 (6.1)	1 (4.0)	1
Postop PROMIS-physical health, mean ± SD	40.5 ± 7.4	43.3 ± 10.6	0.339
Postop PROMIS-mental health, mean ± SD	46.0 ± 7.9	49.2 ± 11.5	0.163
Change in pre-postop PROMIS-physical health, mean ± SD	8.0 ± 8.0	5.2 ± 10.1	0.258
Change in pre-postop PROMIS-mental health, mean ± SD	4.9 ± 10.0	4.4 ± 9.4	0.919
MCID PROMIS-physical health, n (%)	22 (66.7)	15 (60.0)	0.805
MCID PROMIS-mental health, n (%)	14 (42.4)	13 (52.0)	0.647
Follow-up, months, mean ± SD	8.0 ± 5.8	10.9 ± 6.4	0.044

## Discussion

Our findings demonstrate that patients undergoing lumbar fusion for DLS largely present in a similar manner and experience similar clinical and patient-reported outcomes when stratified using the CARDS classification system. While rates of interbody fusion varied across CARDS groups, the idiosyncratic results observed suggest the role of additional patient factors beyond those measured by CARDS and the fact that surgeon discretion continues to influence decisions regarding fusion techniques. When examining the two most common CARDS classes (B and C), significantly lower levels of postoperative physical and mental health were observed among the CARDS C patients, although both groups experienced similar improvement in outcomes. Finally, the finding of minimal differences in any characteristics and outcomes between CARDS C(a) and CARDS C(b) patients indicates that no further subclassification of this group is warranted.

In alignment with our results, prior studies have found that the baseline clinical characteristics of patients are largely similar across CARDS groups. In a study of 78 surgical patients with L4-L5 DLS, Sobol et al. found no significant differences in age, BMI, or baseline Oswestry Disability Index (ODI), Short Form-12 (SF-12) mental or physical components, or visual analog scale (VAS) leg pain across CARDS groups [14]. However, significant differences in VAS back pain were observed, with CARDS D patients presenting with higher levels of pain than the other CARDS groups [14]. This aligns with our finding of a greater proportion of these patients (40%) reporting axial-only pain in comparison with other groups. Another study by Karamian et al. involving 188 patients with L4-L5 DLS undergoing lumbar fusion observed similar trends in patient presentation [11]. At baseline, the authors found that the patients were similar in age, sex, BMI, smoking history, and symptom duration across CARDS groups, while rates of worker’s compensation did vary across the population [11]. While our findings are in alignment with those of previous studies, the current study adds to our understanding of patient presentation by demonstrating a lack of significant differences in baseline PROMIS physical and mental health scores, symptom types, and duration across CARDS groups.

The utility of the CARDS system for guiding surgical planning, and the performance of interbody fusion specifically, remains an important topic of investigation. In the current study, we observed higher rates of interbody utilization in CARDS B and D (60%) patients than those in the CARDS A (33%) and CARDS C (19%) classes. However, this finding is limited by the smaller number of CARDS A (n=3) and CARDS D (n=5) patients in our cohort. In comparison with the Karamian et al. study, the overall utilization of interbodies was similar, as 33% of patients in our study underwent PLF+TLIF/PLIF, while 26.6% of patients in the former study underwent PLF+TLIF [11]. However, the distribution of interbody use in our study differed from the Karamian study, as PLF+TLIF was performed in 13% of CARDS A, 19.2% of CARDS B, 28.2% of CARDS C, and 60% of CARDS D patients [11], suggesting that additional factors beyond those captured by the CARDS system continue to influence decisions regarding interbody use.

The effects of interbody fusion on outcomes across CARDS groups were evaluated in a study of 1056 patients undergoing single-level PLF or PLF+TLIF by Issa et al. [15]. While the authors found no differences in the incidence of revisions, complications, or readmissions between the surgical approaches, CARDS A patients undergoing PLF alone were less likely to achieve a minimal clinically important difference for back pain [15]. Furthermore, PLF+TLIF was independently predictive of greater VAS leg improvement at one year postoperatively in CARDS A patients [15]. However, no significant differences in patient-reported outcome measures (PROMs) were observed across the other CARDS classes in the univariable or multivariable analyses, leading the authors to conclude that while CARDS A patients appear to benefit from TLIF, no additional benefit of interbody placement exists in CARDS B or C patients [15]. Given that interbody placement can provide enhanced disc space restoration and indirect neuroforaminal decompression [15,22,23], the benefit of this approach among CARDS A patients is logical. Unsurprisingly, overall trends in the performance of interbody fusions are variable across CARDS groups in the current study and others given the heterogeneous nature of DLS. Hence, while the CARDS classification provides a useful framework for stratifying DLS patients and assessing the effectiveness of various surgical approaches across differing radiographic presentations, further research is required to enhance its utility for preoperative planning.

In the current study, minimal differences in clinical and patient-reported outcomes were observed across CARDS groups. The most notable findings from our study were variable levels of postoperative physical function across CARDS classes and lower levels of postoperative physical and mental health in CARDS C vs. CARDS B patients. However, this may be a statistical anomaly rather than a finding of clinical significance given that similar improvements in PROMIS scores and rates of MCID achievement were observed between groups. These results are in alignment with three prior studies, which all reported similar levels of improvement in ODI, SF-12 mental, and VAS back and leg scores at one year postoperatively across CARDS groups [11,14,15]. However, others have described greater variability in patient-reported outcome improvements across groups, and greater improvement in SF-12 physical and VAS back pain scores in the CARDS D population specifically [15,19]. Although the current study did not assess radiographic outcomes, the findings of prior studies suggest the CARDS classification system may hold greater prognostic ability in this domain. Karamian et al. reported significant differences across CARDS groups concerning changes in lumbar lordosis (LL), which ranged from -1.63 degrees in CARDS A patients to 3.19 degrees in CARDS D patients, and changes in LL-pelvic incidence (PI) ranging from -3.70 degrees in CARDS D to 2.09 degrees in CARDS B patients [11]. While the relationship between postoperative radiographic and patient-reported outcomes in patients undergoing fusion for DLS has been previously described [11,24,25], further studies are warranted to evaluate whether the magnitude of variation in postoperative spinopelvic parameters across CARDS groups translates into differences in long-term functional status and quality of life.

A final notable finding of the current study was the lack of significant differences in presentation and outcomes of CARDS C patients when sub-stratified into the C(a) (5-9 mm of anterior translation) and C(b) (9+ mm of anterior translation) groups. We chose to perform this subgroup analysis post-hoc based on the relatively high levels of translation and variability observed within the CARDS C group (8.8 mm), which encompasses all patients with anterior translation less than 5 mm. As CARDS C was the most common presentation in the current (64% of patients) and prior studies [11,14,15,18], we wished to evaluate whether a subclassification of this group would enhance the system’s clinical utility. Based on the negative results of this subgroup analysis, we propose that no further refinement of the CARDS C classification is warranted. Continued utilization of the CARDS classification system in its current design is recommended to preserve its simplicity, which enhances the potential for continued uptake into clinical practice.

This study has several limitations. Firstly, as a single-institution, observational study, our population may not be representative of DLS patients broadly. Second, the decisions to offer and proceed with fusion surgery are inherently influenced by surgeon and patient discretion and may introduce selection bias. Third, while we examined basic demographics and comorbidities and found minimal differences across CARDS groups, it is likely that unmeasured confounding factors influenced our results. Fourth, the classification of patients into the various CARDS groups introduces some degree of subjectivity from the reviewer. However, prior studies have demonstrated that the classification system has high inter-observer reliability, mitigating the risk of bias to some extent [4,14,16]. Finally, the relatively small sample size of the study presents the potential that our largely negative findings are a result of type II error. Replication studies using larger sample sizes as well as meta-analyses are warranted to confirm our results. Despite these limitations, we believe the results of the current study are valuable given the relative paucity of evidence evaluating the CARDS classification system.

## Conclusions

Patients undergoing PLF for DLS present with similar demographic and clinical characteristics, and experience similar clinical and patient-reported outcomes when stratified using the CARDS classification system. While some deviations in postoperative physical function were observed, the level of improvement was similar across CARDS groups, suggesting that surgical intervention can be effective for various radiographic presentations of degenerative spondylolisthesis. Further research is warranted to assess the utility of the CARDS classification system in preoperative planning and surgeon decision-making.
